# Current Indications for Seed-Marked Axillary Lymph Node Dissection in Breast Cancer

**DOI:** 10.3390/cancers17101682

**Published:** 2025-05-16

**Authors:** Adolfo Loayza, Elisa Moreno-Palacios, Laura Frías, Ylenia Navarro, Marcos Meléndez, Covadonga Martí, Diego Garrido, Alberto Berjón, Alicia Hernández, José I. Sánchez-Méndez

**Affiliations:** 1 Breast Unit, Department of Gynecology and Obstetrics, Hospital Universitario La Paz, 28046 Madrid, Spain; elisa.moreno@salud.madrid.org (E.M.-P.); lfrias@salud.madrid.org (L.F.); marcos.melendez@salud.madrid.org (M.M.); covadonga.marti@salud.madrid.org (C.M.); joseignacio.sanchez@salud.madrid.org (J.I.S.-M.); 2 Radiodiagnostics Service, Hospital Universitario La Paz, 28046 Madrid, Spain; ylenia.navarro@salud.madrid.org (Y.N.); diego.garrido@salud.madrid.org (D.G.); 3 Department of Pathology, Hospital Universitario La Paz, 28046 Madrid, Spain; alberto.berjon@salud.madrid.org; 4 Faculty of Medicine, Universidad Autónoma de Madrid, 28029 Madrid, Spain; ahernandezg@salud.madrid.org; 5 Gynaecology Department, La Paz University Hospital, 28046 Madrid, Spain; 6 Molecular Pathology and Therapeutic Targets Group, La Paz University Hospital (IdiPAZ), 28046 Madrid, Spain

**Keywords:** axillary recurrence following breast cancer, seed-marked axillary lymph node dissection, targeted axillary dissection, seed marking

## Abstract

**Purpose:** Marker placement in a pathological node improves extirpation rates in breast cancer cases with limited axillary involvement. Our goal was to assess the current indications for seed-marked axillary lymph node dissection (SMALND). **Methods:** We conducted a descriptive observational study, including 93 patients with cN1 breast cancer treated between January 2019 and December 2023. Seed placement was performed under ultrasound guidance, days before the procedure. Intraoperative detection was achieved using a probe, and resection was confirmed radiologically. **Results:** The primary indication was post-neoadjuvant therapy (72 patients: 60 for chemotherapy and 12 for hormone therapy), followed by initial surgery (14) and a single axillary recurrence (8). The extirpation rate of the marked axillary lymph node was 100%. In targeted axillary dissection (TAD), the concordance rate between the sentinel node and the marked axillary node was 85%. In the 12 cases of initial surgery, axillary lymphadenectomy was avoided because the marked node matched the sentinel node and was the only one involved. **Conclusions:** The use of seeds was proven to be highly useful in axillary surgery, both in cases of negativization following neoadjuvant therapy and in those with low axillary involvement or a single axillary recurrence.

## Simple Summary

Marker placement in pathological lymph nodes can improve resection rates in breast cancer with limited axillary involvement. Our goal was to assess in which cases seed-marked axillary lymph node dissection (SMALND) is indicated. Our findings concluded that the most prevalent indication was after neoadjuvant therapy, followed by initial surgery and axillary recurrence. The extirpation rate of the marked axillary node was 100%, and in the case of targeted axillary dissection (TAD), the rate of concordance between the sentinel node and the marked axillary node was 85%. The use of seeds was proven to be highly useful in neoadjuvant therapy and in cases of primary surgery with low axillary involvement or a single axillary recurrence.

## 1. Introduction

Breast cancer is the most commonly diagnosed malignancy and the leading cause of cancer-related death among women [1].

Axillary node involvement is one of the most important prognostic factors. As lymph node involvement increases, the survival rate decreases, regardless of the tumor size [2]. Therefore, accurate loco-regional staging is essential for appropriate treatment planning.

Until recently, the standard treatment for axillary disease was lymphadenectomy, regardless of the axillary tumor burden. However, axillary lymphadenectomy is associated with a series of severe complications, such as upper-limb lymphedema, restricted mobility, and decreased quality of life [3,4].

Currently, in the early stages of breast cancer without axillary involvement, the most reliable method for loco-regional staging is sentinel lymph node biopsy (SLNB). The sentinel node (SN) is the first lymph node to receive direct lymphatic drainage from the tumor and serves as a predictor of the involvement in the remaining nodal pathway. When lymph nodes are initially affected, axillary management becomes more complex and requires additional considerations based on specific clinical scenarios.

Regarding axillary involvement and neoadjuvant therapy, neoadjuvant therapy allows for less-extensive breast surgeries when tumors respond adequately. Recently, in cases of limited lymph node involvement (cN1, positive cytology or histology) and clinical–radiological normalization following neoadjuvant therapy, SLNB has been proposed as a possibility, avoiding lymphadenectomy if the non-histological involvement of the sentinel lymph node is demonstrated [5]. However, trials such as ACOSOG Z1071 [6] have reported an unacceptable false-negative rate (FNR), as it did not fall below 10%. This highlighted the need for a new approach to improve sensitivity and reduce the FNR.

In this scenario, ensuring the resection of the previously affected node during SLN is crucial, as it helps reduce the FNR and improves the assessment of residual disease following neoadjuvant therapy [7]. The ACOSOG Z1071 trial concluded that placing a clip in the metastatic node at diagnosis and ensuring its removal during surgery after neoadjuvant chemotherapy reduced the false-negative rate to 6.8%. In contrast, when the affected node was not clipped, the FNR increased to 13.4% [8].

Subsequently, in an attempt to reduce unnecessary lymphadenectomies, the pre-surgical localization and TAD of the affected node with the use of a radioactive seed (iodine-125), clip, and carbon ink was developed [9]. The localization of the pathological axillary node marked with a clip requires the intraoperative involvement of a sonographer, as it does not emit a detectable signal. Regarding the marking of pathological axillary lymph nodes with carbon, there are conflicting data. Some studies report high detection rates, while others indicate rates as low as 82% [10]. Although it is a cost-effective method, there is a risk of carbon diffusion to other lymph nodes and adjacent tissues. Additionally, carbon cannot be visualized before surgery, which limits preoperative planning. Furthermore, there are no standardized protocols regarding the dose or timing of the injection. We currently have the option to use magnetic or radar seeds to this effect.

Other possible scenarios for the use of seeds are the localization of the single metastatic nodes in patients undergoing primary surgery or single-node recurrence resection.

Regarding axillary involvement and primary surgery, in patients with clinically negative axilla who meet the ACOSOG Z-0011 [11] criteria (T1 or T2 tumors with one or two positive sentinel nodes), lymphadenectomy can be avoided. In these cases, an axillary ultrasound could lead to overtreatment, since patients with a cytological or histological diagnosis of axillary involvement will be subjected to an axillary lymphadenectomy, based on some current recommendations. Many of these patients could have been candidates for SLNB if the ultrasound had not been performed. However, it is possible that some of them with axillary involvement and a higher probability of lower axillary tumor burden could be candidates for targeted axillary dissection (TAD) or, similarly, for SMALND plus SLNB [12].

Regarding a single axillary recurrence, in the case of non-palpable axillary recurrence or palpable recurrence that becomes non-palpable after neoadjuvant therapy, marking would ensure removal, regardless of whether or not the lymphadenectomy is completed. Since radiological diagnosis of axillary recurrence is quite rare [13], there are no data on how to mark these lesions. In 2018, our institution introduced the use of magnetic seeds for the localization of non-palpable breast lesions [14] and the pre-surgical localization of the metastatic axillary node following neoadjuvant treatment. In addition, the same method has been used to mark affected lymph nodes in cases of the initial surgical treatment of a single metastatic axillary lymph node.

The main objective of our study was to define the scenarios where SMALND is indicated. The secondary objectives included determining the advantages of SMALND, calculating the seed retrieval rate, assessing the concordance between SNs and seed-marked axillary nodes (ANs), and evaluating the safety of the technique.

## 2. Materials and Methods

### 2.1. Study Design

We designed a retrospective, descriptive observational study including patients diagnosed with breast cancer with limited axillary involvement (cN1). It was approved by the Ethics Committee of our hospital.

We included ninety-three patients treated for breast cancer between January 2019 and December 2023. Every patient was over 18 years and had been diagnosed with breast cancer with limited axillary involvement and underwent SMALND.

The confidentiality of all the study data was respected.

Data from the electronic medical records were obtained, and the following variables were collected: the age of the patient at diagnosis, the anatomic-pathologic diagnosis, the molecular classification, the TNM classification, the number of radiologically affected lymph nodes, the number of marked nodes, the complications during the placement of the seed, the type of neoadjuvant therapy, the clinical–radiological response, the type of breast surgery, whether or not a selective SLNB was performed, the number of sentinel nodes, the result of the SLNB, whether or not a lymphadenectomy was performed, SMALND, the concordance or not of the sentinel lymph node with the marked node, the definitive breast surgery result, the definitive result of the sentinel node if it did not match the marked node, the result of the lymphadenectomy, the number of affected lymph nodes, the total number of nodes obtained, and the pathological TNM stage.

### 2.2. Procedures

Upon suspected axillary involvement, a puncture/biopsy was performed by the Radiology Service. Cytological or histological confirmation of involvement was conducted by the Anatomical Pathology Department. Following approval by the Multidisciplinary Committee, a Hydromark (Devicor Medical Products, Inc., Cincinnati, OH, USA) coil-marker was placed in the lymph nodes in cases requiring primary systemic treatment (chemotherapy or hormone therapy). In neoadjuvant cases, the placement of entry seeds was not performed due to the necessity of confirming a complete radiological response for the subsequent proposal of TAD. The use of coils is considered a more cost-effective alternative, and the detectability of seeds can also be compromised after the neoadjuvant treatment period. Additionally, magnetic seeds can generate artifacts that hinder the evaluation of the radiological response via magnetic resonance imaging.

In cases not requiring neoadjuvant treatment (initial surgery or some cases of a single metastasis), direct placement of the seed was performed.

Both the SMALND and the breast surgery were conducted by one or two gynecologists in the Breast Unit.

The seed placement in the metastatic lymph node was conducted by the breast radiologist of our institution under ultrasound guidance. First, the axillary lymph node to be marked was located in the ultrasound, and the approach was planned. After disinfecting the area, local anesthesia was administered at the access point and in the subcutaneous pathway. After that, a small incision was made in the skin with a scalpel, through which the needle containing the seed was inserted (with gauges ranging between 14G, 16G, and 18G, depending on the seed used). The needle moved forward until its tip was inside the node, and at this time, the safety lock of the needle was removed, and the seed was released. Once released, the needle was removed, and the adequate placement of the seed was verified via ultrasound. Upon completion of the procedure, the correct localization of the seed in the lymph node was radiologically verified.

In cases of TAD, the migration of the radiotracer to the SN and the concordance with the seed-marked AN were checked by means of lymphogammagraphy by the Nuclear Medicine Service.

For the intraoperative localization of the seed-marked lymph node, the corresponding probe was used. At our institution, we can use three types of devices to localize non-palpable lesions: magnetic systems, such as Magseed (Endomag Ltd., Cambridge, United Kingdom) and Sirius Pintuition (Sirius Medical Systems Inc., Amsterdam, The Netherlands), or radar, such as Savi Scout (Merit Medical Systems, Inc., South Jordan, UT, USA). Magseed is the smallest and easiest seed to place, but it requires the use of plastic instruments during surgery. Conversely, Sirius Pintuition is the largest seed, and, from a technical standpoint, its placement is more complex, although it does not require specific materials. Both magnetic seeds generate artifacts in the magnetic resonance imaging (MRI) that complicate the evaluation of the radiological response to the treatment. In contrast, Savi Scout has an intermediate size and does not produce artifacts in the MRI, making it the preferred option when the radiological response can only be assessed through MRI. It has no time limit for detection, although it may not emit a signal at depths greater than 6 cm. Following the resection of the marked lymph node, the presence of the seed in the node was confirmed either macroscopically or radiologically.

In the cases of TAD where a pathologic complete response (PCR) was expected after neoadjuvant treatment and where the intention was to avoid a lymphadenectomy, the lymph nodes were studied intraoperatively with hematoxylin–eosin staining of the sections by the Anatomical Pathology Service. The OSNA (one-step nucleic acid amplification) technique was not employed for nodal staging following neoadjuvant chemotherapy due to the lack of well-defined clinical guidelines for its application in this specific context. In cases of upfront surgery, the nodes were studied by deferred ultrastaging with OSNA. Statistical analyses were performed with the SPSS v15 software (IBM corp., Armouk, USA).

## 3. Results

During the study period, 93 SMALND procedures were performed. The initial radiological study detected 1 node in 67 cases, 2 in 17 cases, and 3 in 9 cases (patients who had received neoadjuvant chemotherapy).

Indications for SMALND were given in 72 cases following primary systemic treatment, including 60 after neoadjuvant chemotherapy and 12 following neoadjuvant hormone therapy; 13 cases of initial surgery; and 8 cases of a single axillary recurrence.

Table 1 shows the TAD results (SMALND plus SLNB) of the cases after neoadjuvant hormone therapy (NAHT). All patients were postmenopausal, and all tumors were classified as luminal T1-3N1.

Table 2 shows the initial surgery cases. Table 3 shows the axillary recurrence cases. The patients ranged in age from 40 to 70 years old, and all tumors were classified as Luminal T1-3N1.

There was an axillary pathological complete response (PCR) in 27 (45%) cases following neoadjuvant chemotherapy (NACT).

Of the 12 cases of neoadjuvant endocrine therapy (NAHT), PCR was found in 1 case (8%).

Of the 13 patients who underwent initial surgery, only one axillary lymphadenectomy was performed because of a second positive unmarked SN.

Of the eight cases of recurrence, only SMALND was performed in five, while an axillary lymphadenectomy was also performed in three.

The detection rate of the marked axillary lymph nodes was 100%, even though the seed was found in the node capsule in five cases.

TAD was performed in 79 cases, and in 66 (84%) of them, the SN matched the marked node.

Magseed was used in 67 (72%) cases; the Savi Scout seed was used in 20 (21.5%); and the Sirius Pintuition seed was used in 6 (6.5%).

## 4. Discussion

In our case studies, the most prevalent indication for SMALND was TAD after neoadjuvant treatment. TAD following neoadjuvant chemotherapy has been well studied. In a systematic review from 2021, the combined analysis showed that the FNR associated with SMALND alone was 6.28%, and in combination with SLNB, it was 5.18%, concluding that both approaches are very accurate in axillary staging in breast cancer patients with positive lymph nodes following NACT [15]. In our case studies, there were 12 cases following NAHT. At our site, NAHT has been a routine practice for a few years [16]. In these cases, it is important to consider that most lymph nodes detected by ultrasound are non-palpable. Additionally, many of the palpable nodes become non-palpable after NAHT despite not achieving PCR. Thus, it is very important to mark these lesions as soon as possible, preferably before initiating systemic treatment. Meanwhile, it is important to consider the low probability of PCR following NAHT when planning TAD with the possibility of avoiding a lymphadenectomy. In our case studies, we only found PCR in one of the 12 patients treated with NAHT. If we consider that the number of affected lymph nodes is significantly higher in patients in whom axillary metastasis was detected by means of ultrasound-guided biopsy [17], and that the number of suspicious lymph nodes in the axillary ultrasound is related to the final axillary burden [18], it is important to select patients who are more likely to have a lower axillary tumor burden to be able to consider the performance of the TAD. Ideal cases are probably those with up to a maximum of two positive nodes [19,20], including smaller, non-lobular, non-G3 tumors, with a low KI67 [12]. Thus, there is a need to prove intraoperatively the existence of a sentinel node without metastatic involvement, different from a pathologic one, to be able to make the decision not to perform an axillary lymphadenectomy. At our institution, we increased the probability of obtaining a sentinel node other than the one marked with the double-marking technique (using the technetium-99m radioisotope and indocyanine green). With the indocyanine green technique, a higher number of sentinel nodes was obtained on average [21]. Of the 12 cases in our case studies, lymphadenectomy could be avoided in 9 patients who had at least an intraoperatively negative node that was different from the marked one. In all cases, a response to NAHT was demonstrated by performing a biopsy at 2–3 weeks post-treatment to analyze ki67 (<10%) and with radiological tests that confirmed both changes in the tumor and the affected lymph nodes.

Every patient treated with NAHT received axillary radiotherapy after surgery.

With respect to the cases treated with initial surgery, with limited axillary involvement, we used the same criteria as for the cases treated with neoadjuvant hormone therapy to decide on the feasibility of TAD. In eleven cases, the imaging tests showed an affected lymph node, while there were two affected lymph nodes in only two cases. In 12 (92%) cases, axillary lymphadenectomy could be avoided, since there was at least one negative sentinel lymph node other than the one marked. All cases presented luminal breast tumors.

Treatment of a single axillary recurrence requires management by a multidisciplinary team, including oncology, radiotherapy, and surgery, among others [22]. Surgical treatment is not well defined. Until two decades ago, the usual treatment of axillary recurrence following a lymphadenectomy was a second rescue lymphadenectomy [23,24]. There is evidence of the safety of a second SLNB in patients with recurrent ipsilateral breast tumors who were previously treated with conservative surgery and who had a negative SLNB [25,26]. However, the performance of a second SLNB following axillary recurrence after a first SLNB has not yet been considered. In studies on axillary recurrence following SLNB, the usual treatment included a lymphadenectomy [27]. Currently, thanks to the availability of the seeds, we can consider SMALND. Of the eight cases in our study, four had a previous ipsilateral axillary AL, where only the affected lymph node was removed, avoiding a second rescue lymphadenectomy. In two cases with a history of a negative SLNB, given the lack of evidence of the safety of a second SLNB, a lymphadenectomy was performed in addition to the seed-marked ALND. In case number 4, with a history of AL, a rescue AL was performed, given the history of the incomplete initial AL. In case 3, only an SMALND was performed due to metastatic progression.

In terms of potential side effects, SLND and SMALND are equivalent. The American College of Surgeons Oncology Group trial Z0011 previously demonstrated that lymphedema occurs more frequently after SLND combined with ALND [28].

No complications have been recorded related to the seed placement or to carrying it for a time period until the surgery.

The localization and removal rate of the seed and the marked lymph node was 100%, although in five cases, the seed was localized next to, and not inside, the node. These results are similar to those reported in the studies using modern seeds to mark the affected nodes [29,30].

Of the cases in which TAD was performed (SLNB + SMALND), in 84%, the SN matched the marked node. In a study with 81 patients where Magseed was used as a marker (the most widely used marker in our study), the general concordance rate between the marked lymph node and the sentinel lymph node was 81.5%. [30]. These results are consistent with the expected false-negative rate of SLNB following chemotherapy [31].

## 5. Conclusions

In the event of axillary involvement, the most prevalent indication for SMALND was following primary systemic therapy (PST) in the context of TAD, although we observed increased indications in cases of initial surgery or a single axillary recurrence.

The implementation of SMALND can reduce the number of axillary lymphadenectomies in the three scenarios described, mainly for primary surgeries with a low axillary burden and in the excision of affected lymph nodes following PST.

According to our findings, the localization and removal rate of the seed-marked lymph nodes was 100%. Complications related to the placement or the presence of the seeds within the pathological nodes were null, and the concordance rate between the seed-marked lymph nodes and the sentinel nodes (SNs) was high (85%).

## Figures and Tables

**Table 1:** Targeted axillary dissection after neoadjuvant hormone therapy.

Age	Tumor Phenotype	Tumor Staging	Affected Lymph Nodes in Image	Marked Lymph Nodes	Number of SNs	SMAN Matches SN	Non-Concordant SN, Intraoperative +	Non-Concordant SN, Intraoperative −	Positive Lymph Nodes in AL	Total Lymph Nodes in AL	SMAN Result
62	L B	T2N1	2	2	4	Yes	0	3	0	0	Negative
71	L A	T3N1	1	1	2	Yes	1	0	3	11	Positive
71	L A	T2N1	1	1	1	No	0	1	0	0	Positive
56	L B	T2N1	1	1	1	No	0	1	0	0	Positive
62	L B	T1N1	1	1	0	Yes	0	0	0	0	Positive
61	L B	T2N1	2	2	2	No	2	0	8	12	Positive
74	LA	T2N1	1	1	3	Yes	1	1	0	9	Positive
80	L A	T2N1	1	1	3	Yes	1	1	0	0	Positive
63	L B	T2N1	1	1	5	Yes	2	2	0	0	Positive
64	L A	T2N1	1	1	3	Yes	0	2	0	0	Positive
72	L A	T2N1	1	1	2	Yes	0	1	0	0	Positive
73	L A	T2N1	2	1	3	Yes	1	1	0	0	Positive

Acronyms: L: luminal; SN: sentinel node; SMAN: seed-marked axillary node; AL: axillary lymphadenectomy. Table 1 shows TAD results (SMALND plus SLNB) of cases following neoadjuvant hormone therapy (NAHT).

**Table 2:** Cases treated with initial surgery.

Age	Tumor Phenotype	Tumor Staging	Affected Lymph Nodes in Image	Marked Lymph Nodes	Number of SNs	SMAN Matches SN	Non-Concordant SN, Intraoperative +	Non-Concordant SN, Intraoperative −	Positive Lymph Nodes in AL	Total Lymph Nodes in AL	SMAN Result
**47**	**L B**	**T2N1**	1	2	2	Yes	1	0	0	9	Positive
**56**	**L B**	**T2N1**	2	2	3	Yes	0	2	0	0	Positive
**70**	**L B**	**T1N1**	1	1	2	Yes	0	1	0	0	Positive
**83**	**L B**	**T2N1**	1	1	2	Yes	0	1	0	0	Positive
**40**	**L B**	**T1N1**	1	1	2	Yes	0	1	0	0	Positive
**43**	**L B**	**T3N1**	1	1	3	Yes	0	2	0	0	Positive
**63**	**L B**	**T1N1**	1	1	2	Yes	0	1	0	0	Positive
**47**	**L B**	**T1N1**	1	1	3	Yes	1	1	0	0	Positive
**40**	**L A**	**T2N1**	1	1	3	Yes	0	2	0	0	Positive
**49**	**L B**	**T1N1**	2	1	4	Yes	1	2	0	0	Positive
**48**	**L B**	**T1N1**	1	1	3	Yes	0	2	0	0	Positive
**47**	**L A**	**T2N1**	1	1	3	No	1	2	0	0	Positive
46	L B	T2N1	1	1	3	Yes	0	2	0	0	Positive

Acronyms: L: luminal; SN: sentinel node; SMAN: seed-marked axillary node; AL: axillary lymphadenectomy.

**Table 3:** Cases of axillary recurrence.

Case	Year of Primary Cancer Diagnosis	Initial treatment	Years Until Relapse	Treatment of Relapse
1	2013	Mastectomy plus AL	9	SMALND
2	2015	Lumpectomy plus AL (3/10)	7	SMALND
3	2016	Mastectomy plus SN (Neg)	4	SMALND only due to metastatic progression
4	2016	Mastectomy plus AL (2/4)	6	SMALND plus rescue AL
5	2017	Lumpectomy plus AL	4	SMALND
6	2017	Mastectomy plus SN (not migrated)	6	SMALND plus AL
7	2018	Lumpectomy plus AL (5/13)	5	SMALND
8	2022	Lumpectomy plus SN (Neg)	1	SMALND plus AL

Acronyms: SN: sentinel node; SMALND: seed-marked axillary node dissection; AL: axillary lymphadenectomy.

## Data Availability

This study was based on real-world patient data, including demographics and comorbidity factors that cannot be communicated due to patient privacy concerns.
